# Case report of eventration of diaphragm due to an unknown febrile illness causing phrenic nerve palsy and other multiple nerve palsies

**DOI:** 10.1016/j.amsu.2020.04.003

**Published:** 2020-04-25

**Authors:** Pradhan P, R.M. Karmacharya, S Vaidya, A.K. Singh, P Thapa, P Dhakal, S Dahal, S Bade, N Bhandari

**Affiliations:** Department of Emergency Medicine, Kathmandu University School of Medical Sciences, Dhulikhel Hospital, Nepal

**Keywords:** Acquired eventration, Case report, Eventration, Nepal, Phrenic nerve palsy

## Abstract

**Introduction:**

Diaphragmatic eventration can be congenital or acquired. Diagnosis is delayed due to no symptoms or very mild ones and is generally done by imaging modalities. This condition is managed by plication of the affected part of diaphragm by various surgical approaches.

**Presentation of case:**

A forty seven years lady presented with one year long history of abdominal pain, bloating and fullness after meals who was being treated in line of peptic acid disorder. She had developed bilateral foot drop after typhoid fever at seventeen years of age. Clinical examination and imaging with chest x-ray, chest ultrasound and computed tomography scan suggested eventration of left hemidiaphragm. Plication of eventration of left hemidiaphragm was done via mini thoracotomy of the left thorax. There were no postoperative complications and she was discharged on the sixth postoperative day.

**Discussion:**

Acquired eventration of diaphragm is commonly due to traumatic phrenic nerve palsy but rarely can be associated with a history of infection causing nerve palsies. Thoracic ultrasound is an emerging modality for diagnosis supporting X-rays and CT Scans. Plication of eventration with minimally invasive techniques has less number of hospital stay and less pain compared to open approaches.

**Conclusion:**

Non-traumatic diaphragmatic eventration due to acquired phrenic nerve palsy following an unknown febrile illness is a rare case to be reported in Nepal. The aim of treatment is expansion of intra-thoracic space which is done by plication of the diaphragm.

## Highlights

• Acquired eventration of diaphragm is commonly associated with traumatic phrenic nerve palsy.
• Non-traumatic acquired eventration of diaphragm can occur following infections that cause nerve palsy.
• Plication of eventration is surgical treatment of choice which has various approaches.

## 1 Introduction

Diaphragmatic eventration is abnormal elevation of diaphragm with normal peripheral attachment. It can be either congenital or acquired [1,2]. Acquired cases occur after trauma to phrenic nerve (mechanical or surgical), compression by space-occupying lesion in thorax, multiple infectious or inflammatory conditions damaging phrenic nerve [2,3]. Diagnosis of diaphragmatic eventration is usually accidental due to its asymptomatic nature or due to mild nature of symptoms. When symptomatic, patients present with shortness of breath or non-specific gastrointestinal symptoms like epigastric pain, burning sensation, regurgitation, nausea, belching and fullness of abdomen [4,5]. Radiological diagnosis with X-ray, Ultrasound, CT scan or MRI is done which may need diagnostic laparoscopy to confirm the diagnosis [[1], [2]]. Plication is done with various approaches for the management of the condition [4,[6], [7], [8], [9]]. This work is reported in accordance with SCARE Criteria [10].

## 2 Case presentation

A 47 years lady presented with abdominal distension, pain and bloating after meals. She had the symptoms since one year and was managed as peptic acid disorder by her physician. She had no history of cough, shortness of breath or chest pain. In past medical illness, when she was 17 yrs of age she was diagnosed with typhoid fever following which she had sudden onset bilateral foot drop. She gives history of multiple fall injuries due to the weakness which resolved on its own. There were no other comorbidities and no past surgical history.

**Fig. 1:**
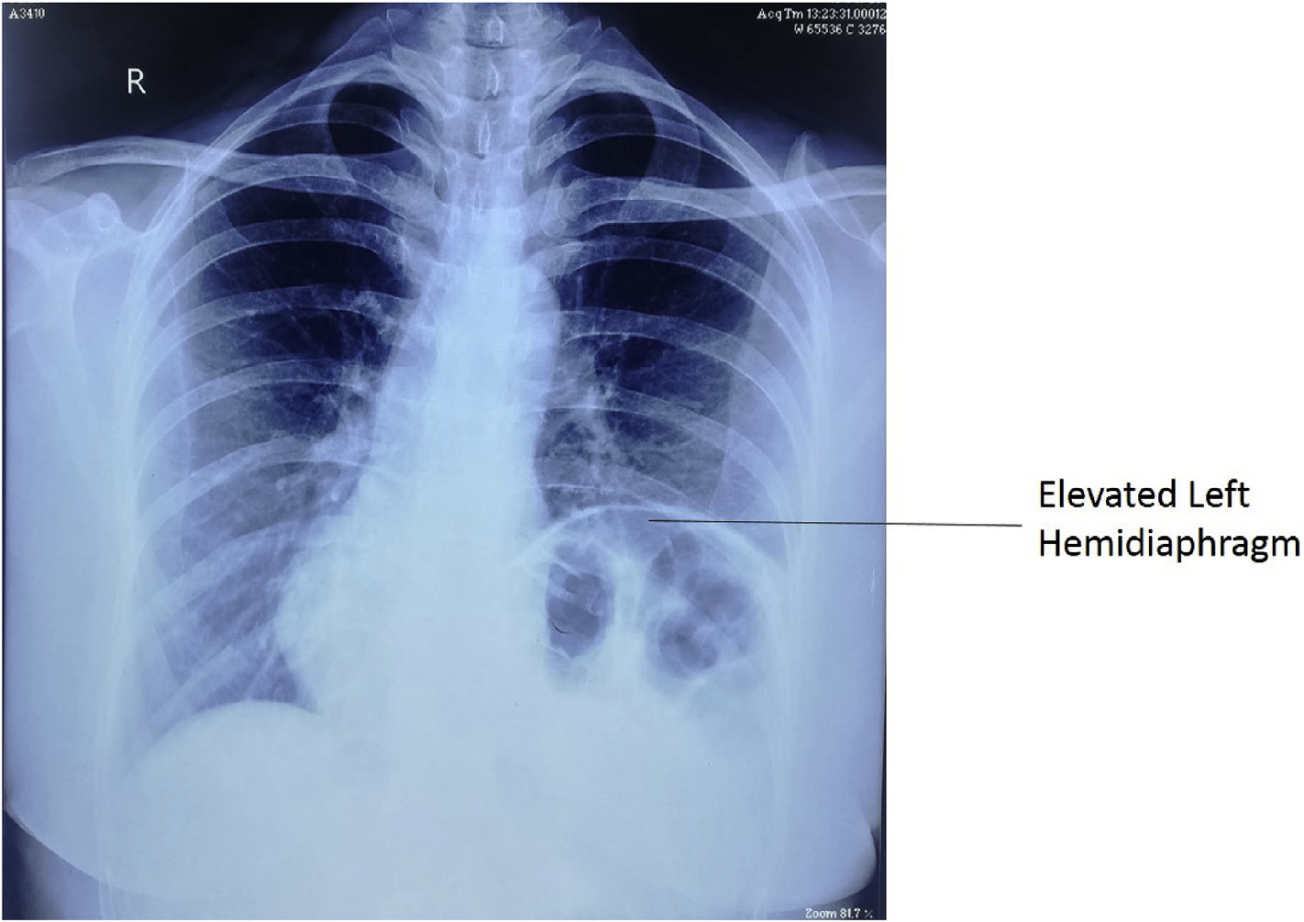
Chest X-ray showing elevated left hemidiaphragm up to 4th rib with normal gas shadow of bowel underneath and mediastinal shift towards the right side.

**Fig. 2:**
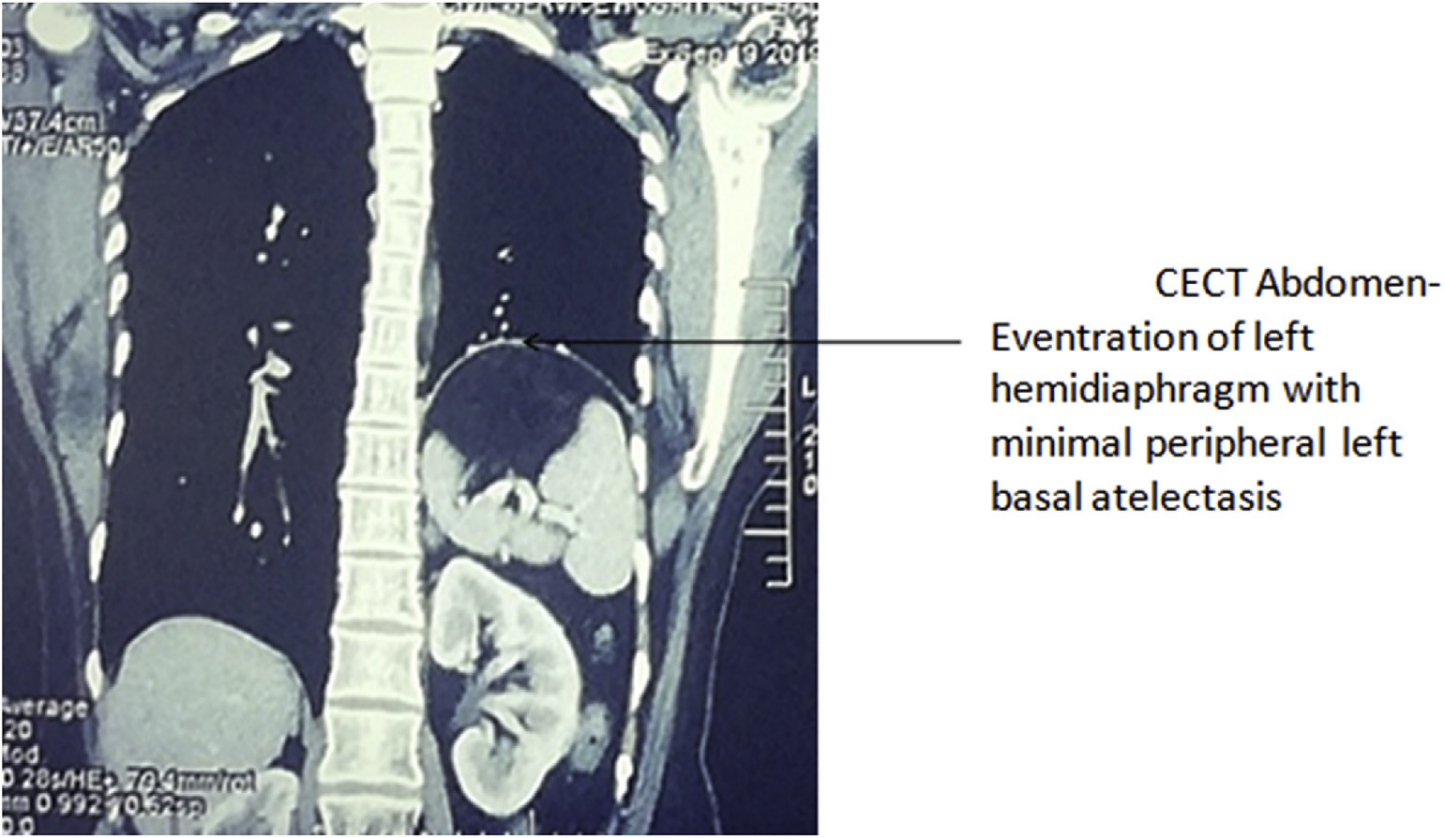
CECT Abdomen-Eventration of left hemidiahragm with minimal peripheral left basal atelectasis.

Her general physical examination was normal but her power of dorsiflexion of bilateral foot was 4/5. Chest examination revealed decreased movement in left infra-mammary, infra-axillary, and infra-scapular areas. Vocal fremitus was decreased on the left side. Breath sounds were decreased in the left infra-mammary, infra-axillary, and infra-scapular areas with dull note on percussion starting from left 4th intercostal space. Bowel sounds were heard from left 5th intercostal space. Other systemic examination and routine laboratory investigations were within normal limits. Chest X-ray (Fig. 1) showed elevated left hemidiaphragm up to 4th rib with normal gas shadow of bowel underneath. Ultrasound showed paradoxical and limited movement of the left hemidiaphragm with breathing. CT abdomen (Fig. 2) showed eventration of left hemidiaphragm with minimal peripheral left basal atelectasis and mediastinal shift towards the right side. A diagnosis of eventration of left hemidiaphragm was made and operative management was considered for treatment for which the patient provided an informed consent.

**Fig. 3:**
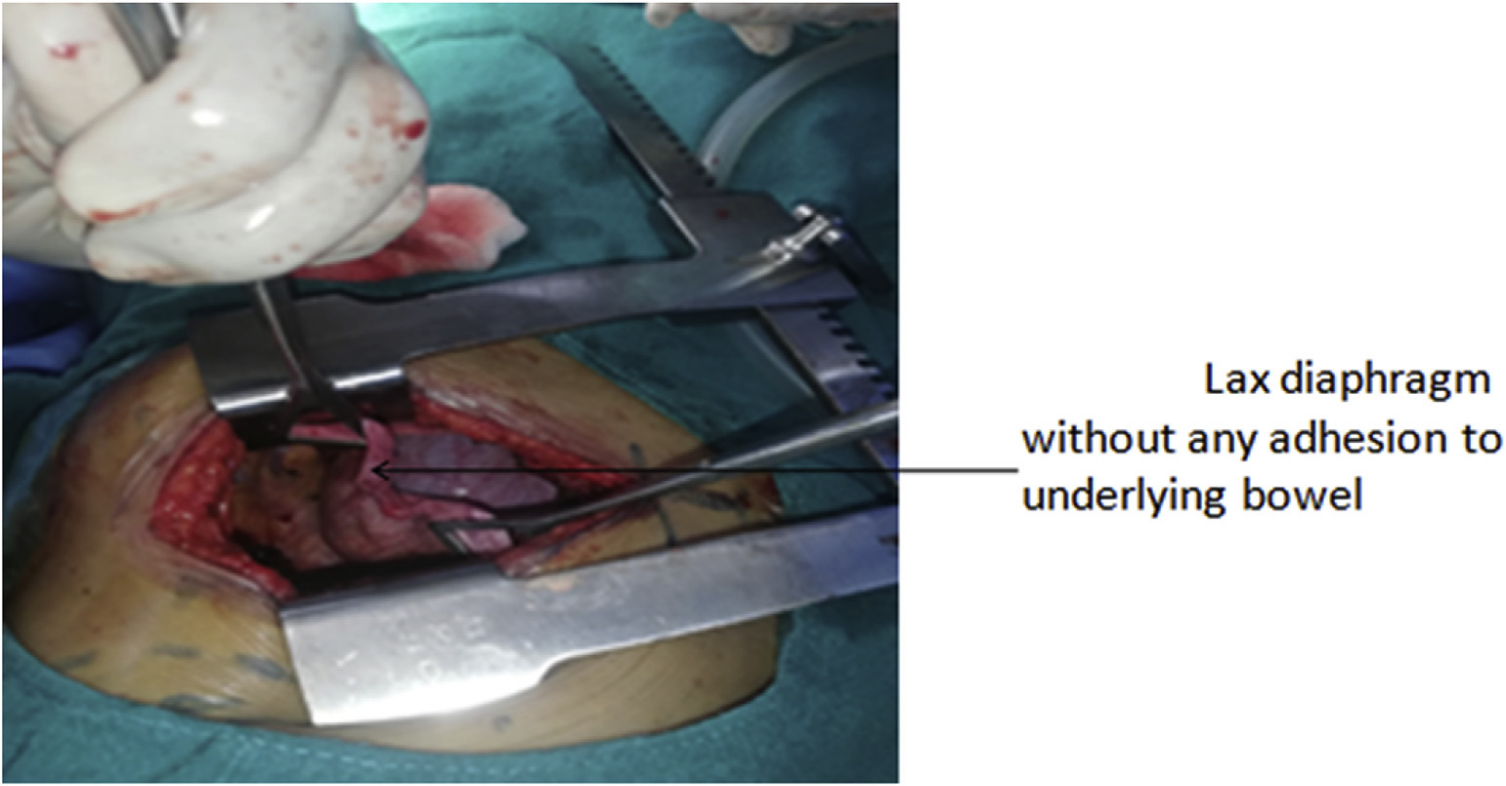
Lax diaphragm without any adhesion to underlying bowel.

**Fig. 4:**
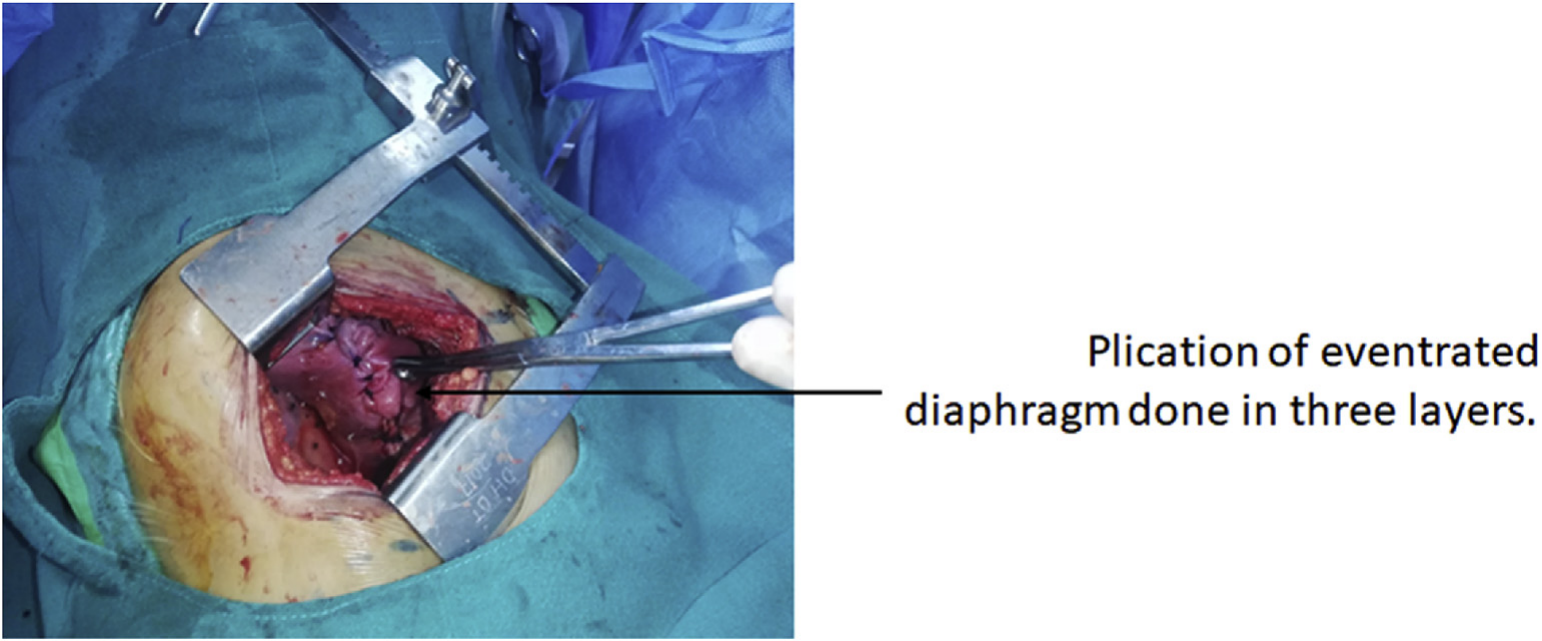
Plication of eventrated diaphragm done in three layer.

Surgical approach was through left mini thoracotomy by two cardiothoracic and vascular surgeons. Mini thoracotomy incision was given in left 7th intercostal space. Eventration of the left diaphragm was visualized with no adhesions with underlying bowel (Fig. 3). Plication of eventration of diaphragm was done in three layers. (Fig. 4). Post procedure full left lung expansion was confirmed. 28 Fr chest-tube drain was inserted in 5th intercostal space and closure was done in multiple layers.

**Fig. 5:**
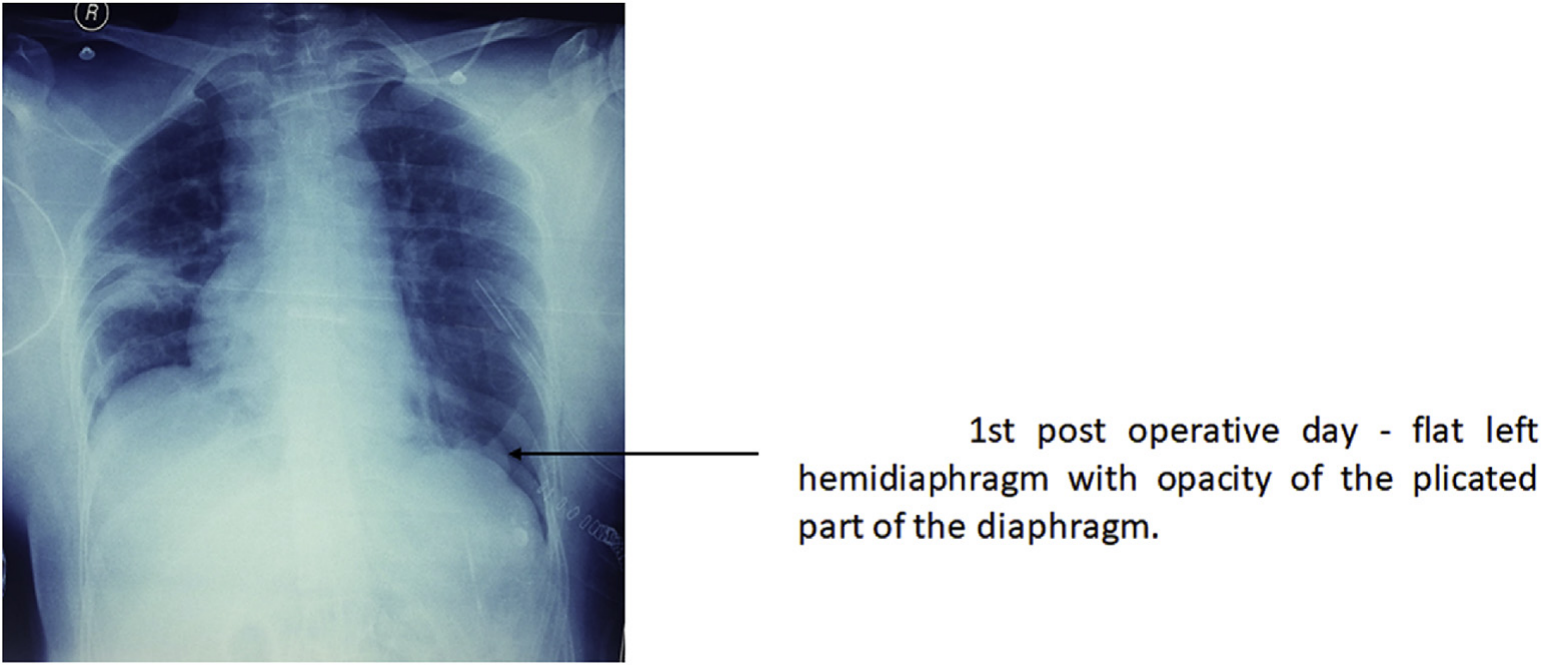
1st post operative day - flat left hemidiaphragm with opacity of the plicated part of the diaphrgm.

In the first postoperative day, chest x-ray, showed flat left hemidiaphragm with opacity of the plicated part of the diaphragm (Fig. 5).

The patient was discharged on the 6th postoperative day. During follow up of a month's duration, she did not have shortness of breath, chest or abdominal pain. Her follow up chest X-ray showed good inflation of left lungs with flattened left hemidiaphragm.

## 3 Discussion

We report a case of a 47 years female who was symptomatic for 12 months with history of early satiety, abdominal fullness and pain. Eventration of diaphragm is a rare pathology usually asymptomatic because the defect is usually small, with some muscle function being preserved and with no compromise of the contralateral diaphragm. If symptomatic, patients usually present with respiratory symptoms such as shortness of breath on lying flat and recurrent respiratory infection. Less commonly, patients may complain of abdominal symptoms, such as vomiting, abdominal pain and fullness [11]. It is more common in males with an average age of 46.5 years [12,13]. Eventration is more common in the left hemidiaphragm as it is with our case [14].

Eventration of diaphragm has been categorized into congenital and acquired according to etiology. Congenital eventration is a developmental defect of one or the entire portion of the central diaphragm with membranous appearance of the muscle with decrease muscle fiber compared to normal. It is present since birth and can present with cardio-respiratory symptoms secondary to lung hypoplasia. Whereas in acquired eventration there is relaxation and loss of contractility of diaphragm with progressive muscular atrophy and distension [15].Symptomatically and radiologically they both appear similar.

Congenital eventration can be associated with other developmental defects like bony abnormalities and recurrent chest infections during childhood [16]. There is complete absence of muscular layer in congenital eventration whereas in acquired ones, muscular layer exists although it may be atrophic [17]. In our case there was no developmental defects and no history of recurrent chest infection during childhood making it less likely to be congenital eventration.

Most common cause of acquired eventration is phrenic nerve palsy due to trauma, either at birth or following thoracic cardiac or pulmonary surgeries. In our case, the probable cause is infectious disease with nerve involvement. The patient gave history of being admitted for enteric fever at 17 years of age following which she suffered sudden onset bilateral foot drop which was considered as a complication of enteric fever. The phrenic nerve palsy could have started since then, may be a milder form and it progressed until she became symptomatic which explains the extremely lax diaphragm suggesting a chronic case. She had no known history of trauma or any surgical procedures. A similar rare case of enteric fever that evolved to guillain barre syndrome leading to phrenic nerve palsy, has been reported in New Delhi, India [3,18]. Some of the other infectious causes of phrenic nerve palsy include arbovirus like West Nile virus, dengue virus and lyme disease [2].

A case report from Nepal shows a 3 months infant with rare combination of congenital diaphragmatic eventration and congenital unilateral lower lip [19]. A case series from Nepal in 2015 reported seven cases of congenital eventration managed with plication [13,19]. A case of 20 years male showing a left-sided diaphragmatic eventration associated with microphthalmia, gastric volvulus, and ipsilateral thyroid agenesis was reported in India [20]. There have been a few uncommon cases of iatrogenic ipsilateral hemidiaphragm paresis reported following cardiotomy, pediatric and adult cardiac surgeries [21] An unusual combination of left recurrent laryngeal and left phrenic nerve palsy in association with thoracic aortic aneurysm was reported by Pradosh K. Sarangi et al., in 2017 [22].

Diagnosis of eventration is based on detailed history and physical examination in symptomatic cases. In asymptomatic cases, it is diagnosed by visualization of elevated affected hemidiaphragm on chest X-rays, mostly an incidental finding. Other modalities like CT, MRI, electromyography and fluoroscopy can be done. Bedside thoracic ultrasound to evaluate the diaphragmatic structure and function is an emerging non-invasive, affordable and reproducible diagnostic test with no risk of ionizing radiation. It has a sensitivity and specificity of 93% and 100% respectively [2,3,23]. In our case, after proper history and examination, chest X-ray confirmed the diagnosis.

Treatment depends on the cause as well as the extent of respiratory compromise due to herniation of abdominal contents into the lung field. Most patients with unilateral paralysis have transient diaphragmatic weakness and complete recovery is expected with time. Sometimes, this weakness can persist for a longer duration despite treatment of the cause. Surgical intervention is required in these cases and also when present as bilateral diaphragmatic paralysis [2,3]. Our patient was symptomatic but she had no respiratory complaints. The goal of treatment of eventration is improving respiratory symptoms by increasing total lung capacity, decreasing paradoxical motion of the diaphragm with decreasing the redundant diaphragmatic surface and decreasing lung compliance allowing intercostal and accessory respiratory muscles with more negative and positive pleural pressure [4,5].Traditional open technique or minimally invasive interventions can be approached from the thorax or abdomen [4]. Open approach is more invasive, painful and requires a longer duration of hospitalization. But laparoscopic and video assisted approaches requires experienced hands and surgical expertise. For rare cases like these, it is quite difficult in our context to get such expertise [12,13]. Hence, in our case, we performed a mini-thoracotomy and 3 layered plication of the diaphragm. The diaphragm was extremely lax, but herniation of abdominal contents were not noticed.

Possible post-surgical complications have been listed such as pneumonia, pleural effusion, pulmonary edema, deep vein thrombosis, pulmonary embolism and risk of abdominal organ injuries. 4,13 A study by Calvinho et al. shows dyspnea as the commonest presentation and chronic pain as the complication associated with plication [24] Our patient was discharged on the 6th post-operative day with no post-surgical complications.

Female patient, gastrointestinal symptoms, no respiratory compromise, acquired phrenic nerve palsy following a febrile illness are some of the important points that make our case unique.

## 4 Conclusion

Non-traumatic diaphragmatic eventration due to acquired phrenic nerve palsy following an unknown febrile illness is a rare case to be reported in Nepal. The aim of treatment is expansion of intra-thoracic space which is done by plication of the diaphragm and is also practiced worldwide for the same motive.

## Ethical approval

NA.

## Funding

None.

## Author contribution

Pradhan P-Writing of the manuscript and involved in the care of the patient.

Karmacharya R.M.- Surgeon involved in the care of the patient and writing of the manuscript.

Singh AK- Surgeon involved in the care of the patient and editor of the manuscript.

Vaidya S-Surgeon involved in the care of the patient and editor of the manuscript.

Thapa P-Writing of the manuscript and involved in the care of the patient.

Dahal S- Writing of the manuscript and involved in the care of the patient.

Dhakal P- Writing of the manuscript and involved in the care of the patient.

Bade S-Writing of the manuscript and involved in the care of the patient.

Bhandari N- Writing of the manuscript and involved in the care of the patient.

## Guarantor

All authors have read and approved the manuscript and accept full responsibility for the work.

## Consent

Written consent was obtained from the patient for publication of this case report and accompanying images.

## Registration of research studies

None.

## Provenance and peer review

Not commissioned or externally peer reviewed.

## Declaration of competing interest

Authors have no conflict of interest to disclose.
